# Supplementary material for: The Potential of Gut Microbiota in Prediction of Stroke-Associated Pneumonia
Source: Brain Sci. 2023 Aug 17;13(8):1217. doi: 10.3390/brainsci13081217 (PMC10452830; doi:10.3390/brainsci13081217)
Supplement: Supplementary file 1 [file brainsci-13-01217-s001.zip › brainsci-2480226-supplementary.pdf]

Supplementary Materials

Contents:

Figure S1. PCoA of gut microbiota between two groups in different distances.

Figure S2. Abundance of gut microbiota OTUs detected in two groups via Venn diagram.

Figure S3. Gut microbiota with significantly different relative abundance between two groups at phylum and family level in Metastats analysis.

Figure S1. PCoA of gut microbiota between two groups in different distances.

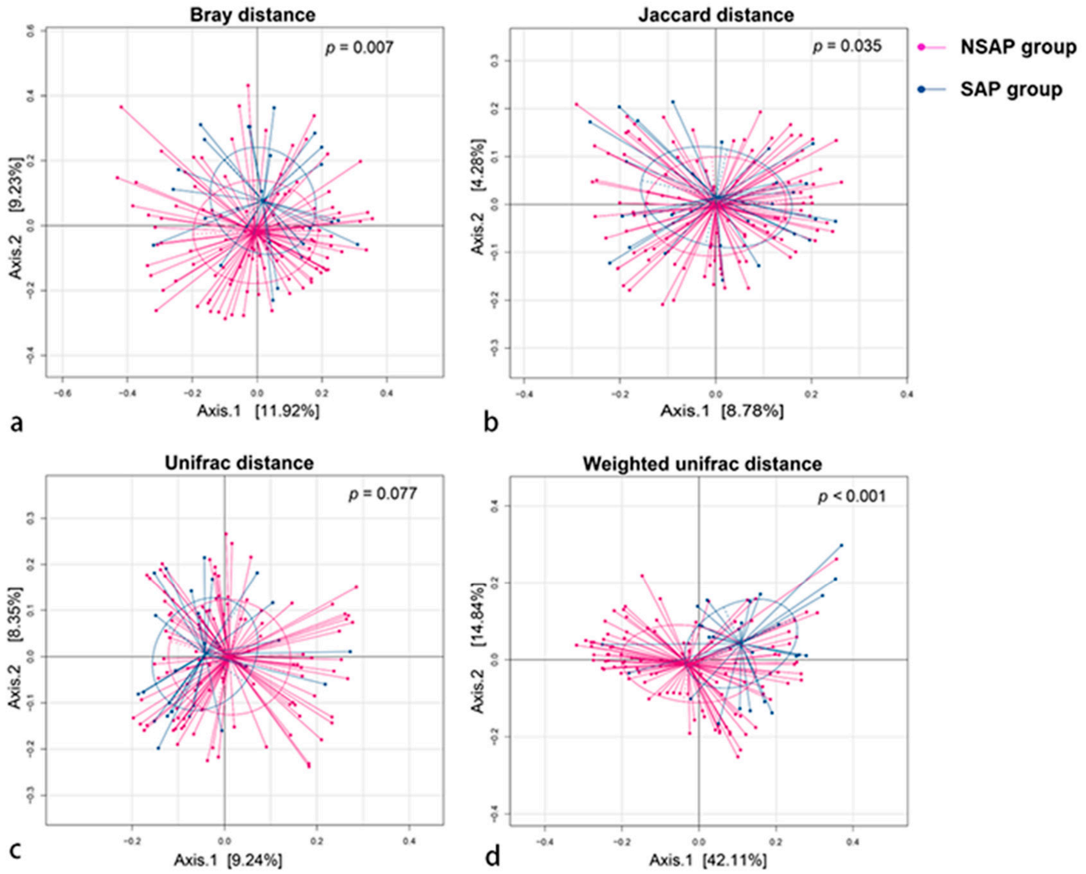

Abbreviation: PCoA, principal coordinate analysis, is the visual result of permutational multivariate analysis of variance (PERMANOVA) according to four distances that showed the differences of Beta diversities between two groups; SAP, stroke-associated pneumonia; NSAP, no SAP group.

Figure S2. Abundance of gut microbiota OTUs detected in two groups via Venn diagram.

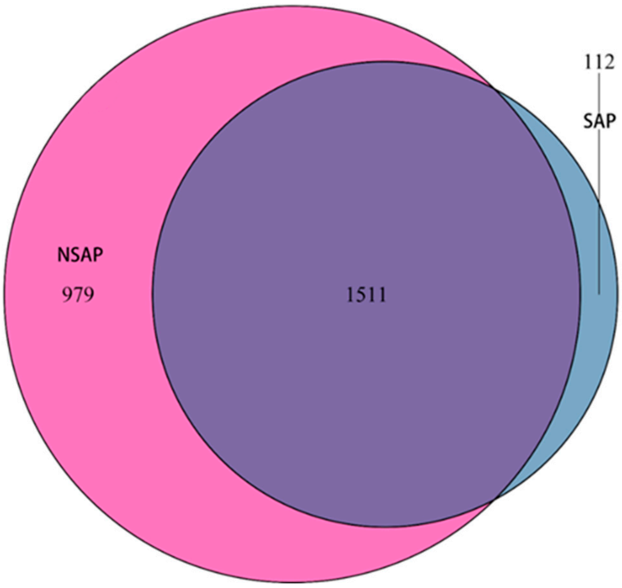

SAP group owned 112 unique OTUs and NSAP group owned 979 unique OTUs. The two groups shared 1511 OTUs.
Abbreviations: OTU, operational taxonomic unit; SAP, stroke-associated pneumonia; NSAP, no SAP group.

Figure S3. Gut microbiota with significantly different relative abundance between two groups at phylum and family level in Metastats analysis.

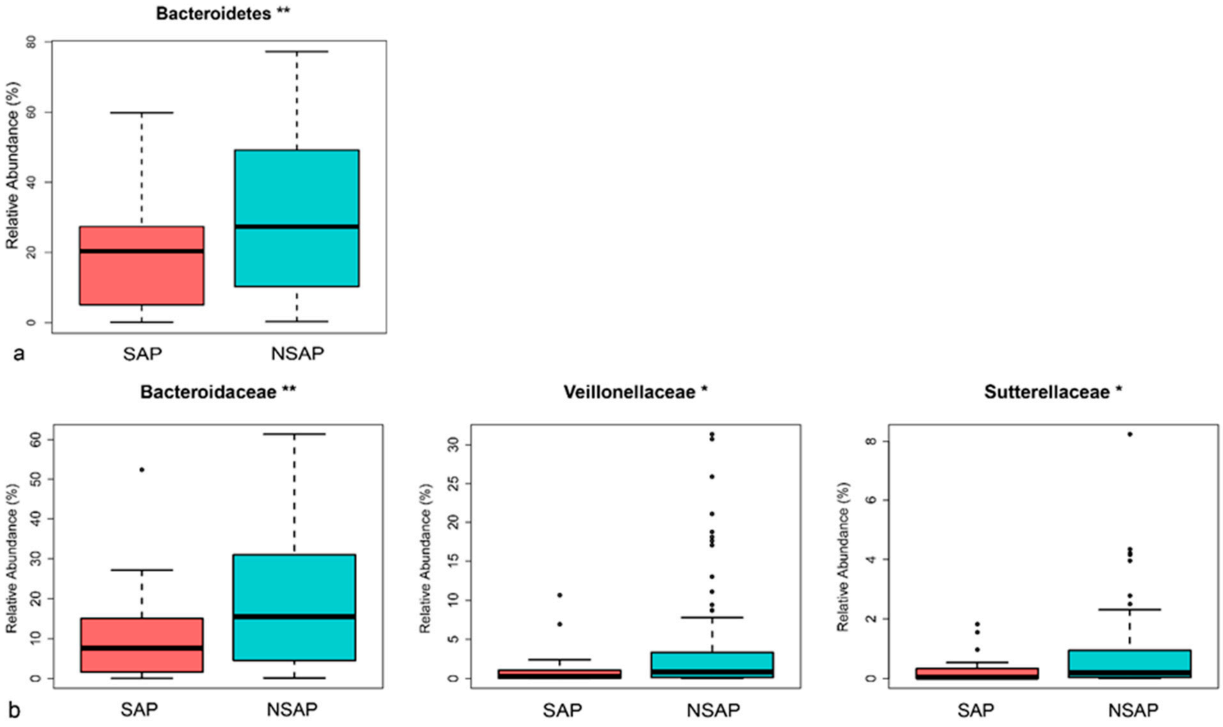

Differential bacteria between SAP patients and no-SAP patients at phylum level (a) and family level (b).

Abbreviations: SAP, stroke-associated pneumonia; NSAP, no SAP group. * $p < 0.05$; ** $p < 0.01$.
